# A Nurse-led Approach to Improving Cardiac Lifestyle Modification in an Atrial Fibrillation Population

**DOI:** 10.19102/icrm.2019.100902

**Published:** 2019-09-15

**Authors:** Kathleen T. Hickey, Elaine Wan, Hasan Garan, Angelo B. Biviano, John P. Morrow, Robert R. Sciacca, Meghan Reading, Theresa A. Koleck, Billy Caceres, Yiyi Zhang, Isaac Goldenthal, Teresa C. Riga, Ruth Masterson Creber

**Affiliations:** ^1^Department of Medicine, Columbia University, New York, NY, USA; ^2^Department of Nursing, Columbia University, New York, NY, USA; ^3^Department of Health Policy and Research, Weill Cornell Medical College, New York, NY, USA

**Keywords:** Atrial fibrillation, nursing, quality of life, telemedicine

## Abstract

Atrial fibrillation (AF) is a major public health problem and the most common cardiac arrhythmia encountered in clinical practice at this time. AF is associated with numerous symptoms such as palpitations, shortness of breath, and fatigue, which can significantly reduce health-related quality of life and result in serious adverse cardiac outcomes. In light of this, the aim of the present pilot study was to test the feasibility of implementing a mobile health (mHealth) lifestyle intervention titled “Atrial Fibrillation and Cardiac Health: Targeting Improving Outcomes via a Nurse-Led Intervention (ACTION),” with the goal of improving cardiac health measures, AF symptom recognition, and self-management. As part of this study, participants self-identified cardiac health goals at enrollment. The nurse used web-based resources from the American Heart Association (Dallas, TX, USA), which included the Life’s Simple 7^®^ My Life Check^®^ assessment, to quantify current lifestyle behavior change needs. Furthermore, on the My AFib Experience™ website (American Heart Association, Dallas, TX, USA), the patient used a symptom tracker tool to capture the date, time, frequency, and type of AF symptoms, and these data were subsequently reviewed by the cardiac nurse. Throughout the six-month intervention period, the cardiac nurse used a motivational interviewing approach to support participants’ cardiac health goals. Ultimately, the ACTION intervention was tested in 53 individuals with AF (mean age: 59 ± 11 years; 76% male). Participants were predominantly overweight/obese (79%), had a history of hypertension (62%) or hyperlipidemia (61%), and reported being physically inactive/not preforming any type of regular exercise (52%). The majority (88%) of the participants had one or more Life’s Simple 7^®^ measures that could be improved. Most of the participants (98%) liked having a dedicated nurse to work with them on a biweekly basis via the mHealth portal. The most commonly self-reported symptoms were palpitations, fatigue/exercise intolerance, and dyspnea. Seventy percent of the participants had an improvement in their weight and blood pressure as documented within the electronic health record as well as a corresponding improvement in their Life’s Simple 7^®^ score at six months. On average, there was a three-pound (1.36-kg) decrease in weight and a 5-mmHg decrease in systolic blood pressure between baseline and at six months. In conclusion, this pilot work provides initial evidence regarding the feasibility of implementing the ACTION intervention and supports testing the ACTION intervention in a larger cohort of AF patients to inform existing AF guidelines and build an evidence base for reducing AF burden through lifestyle modification.

## Introduction

Atrial fibrillation (AF) is a major public health problem and the most common cardiac arrhythmia encountered in clinical practice. In 2017, the estimated prevalence ranged from 2.7 million to 6.1 million in the United States and is projected to grow to 12 million by 2030.^1^ In addition, AF is responsible for approximately 100,000 deaths^2^ and 500,000 hospitalizations annually^2,3^ and is associated with a fivefold increased risk of stroke,^4–7^ a threefold increased risk of heart failure,^8–10^ and an increase in all-cause mortality.^5,11–13^ The burden of AF is currently epidemic and anticipated to grow in prevalence due to an aging population that is living longer with multiple comorbid conditions associated with the development of AF.^14,15^ Moreover, AF has been linked with numerous symptoms such as palpitations, shortness of breath, and fatigue, all of which can significantly reduce health-related quality of life (HRQoL).^16^ Symptom reduction or elimination is a major goal of AF management, which relies on correctly identifying signs and symptoms of AF.^17^ However, AF symptom recognition is challenging, especially in AF populations with multiple coexisting cardiac conditions that exhibit similar symptoms, such as heart failure.

### Treatment and management of atrial fibrillation

Catheter ablation is widely used in clinical practice to treat symptomatic AF.^18^ This treatment uses radiofrequency energy or “freezing” to destroy areas causing AF in the heart.^19–22^ Despite the efficacy of catheter ablation to maintain sinus rhythm, however, 20% to 45% of patients who undergo catheter ablation experience recurrent AF.^23,24^ Electrical cardioversion is another established therapy for the treatment of AF and the maintenance of sinus rhythm. AF recurrence is also common after treatment with this approach, even when patients are receiving additional pharmacological antiarrhythmic therapy to maintain sinus rhythm; about 40% to 60% of patients demonstrate AF recurrence within three months, whereas 60% to 80% experience such within one year after cardioversion.^25,26^ Even a decade after the National Institutes of Health held a special workshop on the prevention of AF and recommended focusing on interventions that prevent AF recurrence,^27^ there remains a paucity of evidence-based, preventative interventions.

### Impact of atrial fibrillation on health-related quality of life

AF has a significant effect on HRQoL, impacting both physical and psychological well-being. Symptoms affecting HRQoL that are commonly associated with AF, such as fatigue, shortness of breath, and palpitations, may also be associated with coexisting cardiovascular conditions like heart failure, making both distinguishing symptoms and management challenging for both patients and providers. Thus, many AF patients, particularly those who are older, fail to recognize and distinguish symptoms associated with AF, ultimately hindering timely treatment and putting them at risk for complications.^28^

### Evidence supporting a healthy cardiac lifestyle reduces atrial fibrillation

It is estimated that a large percentage of AF could be attributed to modifiable risk factors such as hypertension (22%), overweightness/obesity (13%), smoking (10%), and diabetes (3%).^15,29–36^ Among patients with a history of AF, there is a 10% to 29% increase in the risk of postablation AF recurrence for every five-unit increase in body mass index.^37^ Previous research supports the effectiveness of lifestyle modification to improve cardiac health,^38–40^ and nurses are ideally qualified to implement and evaluate such programs in clinical practice.^32,41,42^

Lifestyle modification that results in risk factor reduction in AF patients with a body mass index of greater than 27 kg/m^2^ and at least one other cardiac risk factor (eg, hypertension, diabetes mellitus, smoking, excessive alcohol consumption) has been shown to lead to a drop in AF recurrence and an improvement in patient symptoms.^43^ AF severity, number of AF episodes, cumulative AF duration and recurrence, and medication noncompliance can be reduced when blood pressure, weight, and glucose are well-controlled.^38,39,43–46^ In addition, for patients undergoing treatment by way of catheter ablation for symptomatic AF, a lifestyle-modification intervention resulted in a significant improvement in symptoms after catheter ablation as determined by AF severity score.^47^ The need to address lifestyle modification explicitly highlights a gap in clinical practice and an opportunity for nurses to play a critical role.

Motivational interviewing is grounded in client-centered counseling, cognitive behavioral therapy, and social cognitive therapy.^48^ A motivational interviewing approach is characterized by expressing empathy, developing a discrepancy between belief and behavior, rolling with resistance, and supporting self-efficacy.^49^ One of the goals of motivational interviewing is to help individuals work through the ambivalence present in unhealthy behaviors and to help them verbally express their own reasons for or against change.^49–51^ Several other researchers have demonstrated the benefits of motivational interviewing in changing cardiovascular behaviors and achieving personal cardiac goals.^52^

This evidence supports the need for a nurse-led cardiac lifestyle modification program in clinical practice.^32,41,42^ The aim of the present pilot study was to test the feasibility and preliminary efficacy of a mobile health (mHealth) lifestyle intervention titled “Atrial Fibrillation and Cardiac Health: Targeting Improving Outcomes via a Nurse-Led Intervention (ACTION)” that incorporated the Life’s Simple 7^®^ and My AFib Experience™ web-based tools (American Heart Association, Dallas, TX, USA) to improve cardiac health measures, AF symptom recognition, and self-management by utilizing motivational interviewing.

## Materials and methods

### Study setting

The study was approved by the Columbia University Medical Center (CUMC) Institutional Review Board and took place at the CUMC facility located in northern Manhattan in New York, NY. A trained bilingual (fluent in English and Spanish) research coordinator screened for eligible subjects from the cardiology/electrophysiology service. All participants had a history of paroxysmal or persistent AF. Patients who met the inclusion criteria were enrolled after their primary health-care provider had been made aware of the study protocol and informed consent was obtained.

### Study methods

**Figure 1:**
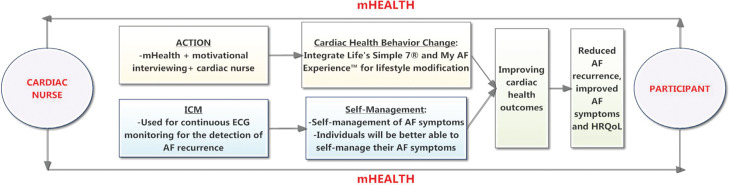
Adapted information–motivation–behavioral skills conceptual model.

**Figure 2:**
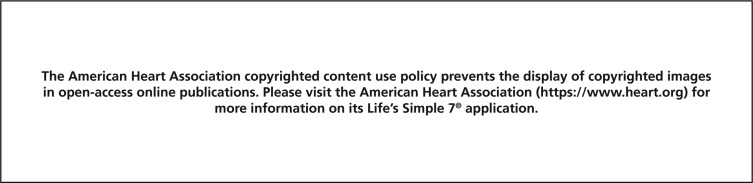
Life’s Simple 7^®^ heart score.

An adapted version of the Information–Motivation–Behavioral Skills Model guided this research **(Figure 1)**.^53^ When information [eg, electrocardiogram (ECG) data, symptoms] and motivation were tailored to improving individual’s health behavior and self-management strategies through the ACTION intervention, these activities were more likely to be integrated into daily routines and potentially result in enhancing symptom recognition and distinguishing symptoms due to AF versus other coexisting conditions. It was thought that this could lead to further improved health changes, (eg, improved self-management of cardiac lifestyle and symptoms) and might potentially improve an individual’s self-recognition of AF and Life’s Simple 7^®^ score **(Figure 2)**.

### Participants

For the purposes of this single-arm pilot study, a convenience sample of 53 participants was included. All study participants continued their clinical management and follow-up as determined by their respective providers.

### Baseline

At baseline, each participant completed the Life’s Simple 7^®^ assessment (American Heart Association, Dallas, TX, USA). All participants selected lifestyle modification areas from the Life’s Simple 7^®^ that they believed they “could act on” or address (eg, physical activity, weight control, eating better) over the six-month study period.

### Intervention

The ACTION intervention used a nurse-led motivational interviewing approach to identify patients’ goals and align lifestyle and self-care management behaviors. This approach included telehealth communication with a nurse guided by the Life’s Simple 7^®^ My Life Check^®^ program (American Heart Association, Dallas, TX, USA) to quantify current lifestyle behaviors as well as use of the My AFib Experience™ web-based resource (American Heart Association, Dallas, TX, USA), which provides a plethora of resources for AF patients to consider. For the purposes of this study, participants utilized the My AFib Symptom Tracker via the My AFib Experience™ website (American Heart Association, Dallas, TX, USA) to capture the date, time, frequency, and type of their AF symptoms. The purpose of the intervention was to improve cardiac health measures, AF symptom recognition, and self-management.

The ACTION intervention was delivered via a secure portal in 30-minute weekly sessions over six months. For each ACTION session, the cardiac nurse and participant connected in a video chat format at a prescheduled date/time via the portal, and an ACTION session was conducted. This time frame was based on previous work that showed that a similar time period is reasonable for a behavioral intervention and for collecting the proposed study endpoints.^43,54–56^

Using a motivational interviewing approach, the cardiac nurse reviewed and discussed lifestyle modification and self-management strategies incorporated by the participant since the previous session. Resources from the aforementioned American Heart Association material were used to guide the sessions, which were personalized to each patient’s individual cardiac goals. Participants completed the My AFib Experience™ Symptom Tracker (American Heart Association, Dallas, TX, USA) during the study period, which allowed them to record the date, time, frequency, and type of any symptoms they experienced related to AF. These symptoms included palpitations/racing heart, shortness of breath, and/or lightheadedness. Participants documented whether symptoms occurred at rest or during exercise as well as their severity and frequency.^57^ This helped guide patients to better self-recognize symptoms and triggers associated with AF versus other coexisting conditions.

### Statistical analysis

Demographic and clinical data are reported as means and standard deviations for continuous variables and as frequencies and percentages for categorical variables. Changes in weight and systolic blood pressure were assessed by paired t-test. Analyses were performed using the SAS version 9.4 software (SAS Institute, Cary, NC, USA). A critical p-value of 0.05 was used for significance in all analyses.

## Results

As part of the present study, we successfully piloted the ACTION intervention in 53 individuals with AF (mean age: 59 ± 11 years; 76% male; 60% white) **(Table 1)**. Almost all participants (98%) reported liking having a dedicated nurse work with them on a biweekly basis via the mHealth portal to help them improve their cardiac health and AF recognition. However, some participants reported that they desired more frequent contact (55%) with the nurse.

Most of the participants (86%) chose increasing physical activity as the primary health goal that they could improve upon, followed by eating a better/healthier diet (65%). Seventy percent of the participants demonstrated an improvement in their weight and blood pressure, as documented in the electronic health record, as well as a corresponding improvement in their Life’s Simple 7^®^ score from baseline to six months. On average, there was a 3-lb ± 13-lb (1.36-kg ± 5.90-kg) decrease in weight and a 5-mmHg decrease in systolic blood pressure from baseline to six months. The overall compliance rate was approximately 65% for the entire cohort. However, for participants with a loop recorder, the compliance rate increased to 76%. On average, participants who received intervention had a one-point improvement in Life’s Simple 7^®^ score over the six-month period. In addition, 70% of the participants improved their Life’s Simple 7^®^ score by one or two points from baseline to six months. Furthermore, 81% of the participants reported that the ACTION intervention enhanced accessibility, eliminated extra in-person visits, and provided personalized cardiac health information in achieving “my health goals,” whereas 86% reported they found the digital ACTION session to be as good as an in-person meeting with the nurse practitioner. Three people did not have Internet access in their home and used the laptop/Wi-Fi of a family member/or neighbor to complete the ACTION sessions. The “human connection” provided by the nurse coupled with the motivational interviewing was noted by participants as the most essential component, and most of the participants (98%) reported that working with a nurse helped keep them to better stay on track with sustaining a healthy lifestyle and with overall AF management.

**Figure 3:**
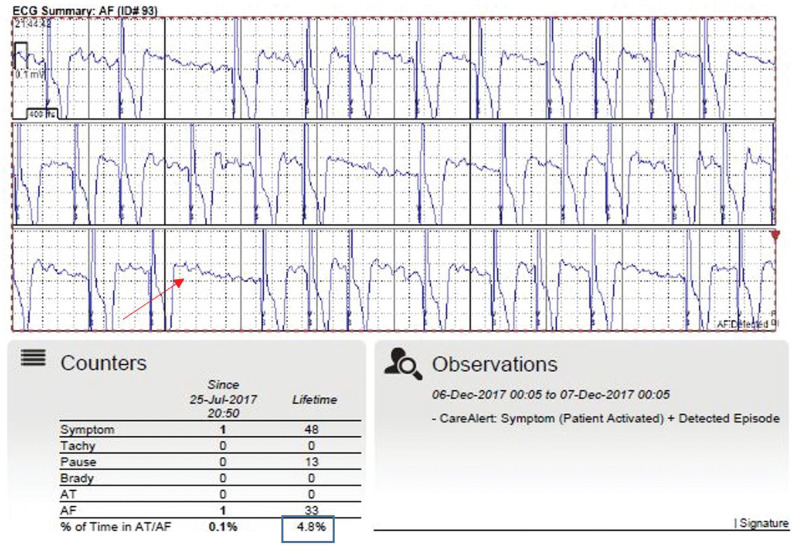
Sample ECG report generated by the ICM. The red arrow highlights AF captured by the ICM, in the setting of active symptoms. The blue box shows the lifetime percent of AF recurrence recorded by the ICM.

Forty-nine percent of the participants had undergone at least one prior cardioversion attempt, 23% had undergone at least one prior ablation, and 17% had undergone both prior ablation and cardioversion. All participants who reported undergoing a prior ablation session had previously also undergone an unsuccessful cardioversion attempt or participated in unsuccessful antiarrhythmic drug therapy. There was no significant change in antiarrhythmic drug therapy from baseline to six months, with 28.3% of patients on antiarrhythmic drugs. Forty-one percent of study participants had an implantable cardiac monitor (ICM) device placed as part of their clinical care following ablation or cardioversion. Regular transmission or transmission initiated by the patient in response to symptoms allowed for the easy transfer of information stored in the device to a central database via the web, where the information was viewed by providers and treatment and management were subsequently provided as needed **(Figure 3)**. In fact, 70% of the participants who self-initiated an ICM transmission did so in the setting of symptoms. The most common symptoms that resulted in an ICM transmission that correlated with AF, atrial flutter, or other arrhythmia being documented on the ECG were palpitations (83%), shortness of breath (81%), and fatigue/exercise intolerance (74%). Most of the participants (91%) reported two or more of these symptoms occurring together, which prompted them to send an ICM transmission to their practitioner for review. Interestingly, 30% of our participants self-reported forgetting to take one or more of their cardiac medications (eg, β-blocker, anticoagulation, diuretic) over the six-month study period and only remembered that they had forgotten to do such upon experiencing symptoms.

## Discussion

The purpose of the present study was to evaluate the feasibility and preliminary efficacy of the ACTION intervention delivered in a single-arm pilot study of AF patients in a large, urban academic medical center. Participants who received the mHealth lifestyle intervention showed improved quantifiable cardiac health measures, such as blood pressure and weight, and AF symptom recognition over a six-month interval. Participants reported that working with a nurse practitioner via a digital portal helped them to stay motivated and focus more on improving their health. In addition, participants with a clinically indicated ICM for AF management were better able to recognize and distinguish their AF symptoms and associated triggers/behaviors.

Lifestyle modification as measured by the Life’s Simple 7^®^ assessment and the My AFib Experience™ website (American Heart Association, Dallas, TX, USA) improves cardiac health by promoting adherence to behavior change, enhancing self-management skills, and empowering patients to actively engage in their care.^58–61^ During the short six-month period of this investigation, there was a one-point improvement in Life’s Simple 7^®^ score. Given a longer study period, it is possible that this improvement would have increased as patients gained more control over their cardiac risk factors and became more engaged in their health care. In addition, while smoking is one of the Life’s Simple 7^®^ measures, there were no active smokers in the cohort. As such, smoking was not a potential area of improvement in terms of Life’s Simple 7^®^ score in this study. Other researchers have found that blood pressure, weight loss, and glucose control reduce AF symptom severity, number of AF episodes, and cumulative AF duration and recurrence.^38,39,43–46^ This evidence supports the need for the incorporation of nurse-led cardiac lifestyle modification programs into clinical practice.^41,42,62^ Other evidence has suggested a nurse-led AF mHealth intervention for AF improves self-management, symptom recognition, HRQoL, and self-recognition of AF.^63^ The compliance rate was similar to that in other studies regarding the use of motivational interviewing for the management of cardiovascular risk factors, which have demonstrated adherence rates ranging from 55.1% to 73.7%.^64,65^ Due to our small sample size, no statistical correlation between compliance and results could be confirmed; however, the increased compliance of the participants with loop recorders is likely an outcome of these participants being more highly engaged with the technology. These individuals became more interested in improving their overall cardiac health by using the patient activator to document arrhythmia symptoms and correlate these symptoms with documented arrhythmias, increasing the likelihood of them remaining compliant.

Regular contact with cardiac nurse practitioners enhanced pharmacologic therapy, including the regulation of β-blocker and antiarrhythmic medications, to better control heart rate and symptoms. Along with lifestyle modification strategies, nurse practitioners often addressed proper anticoagulation and compliance with other medications prescribed for AF and underlying comorbidities. During the sessions, participants often mentioned changes in underlying coexisting conditions that might necessitate a change in medication or treatment, or the participants called members of the research team or the nurse practitioners to report changes in their health status during the study period. This point was essential because, with improper use of or noncompliance with their medication, combined with a lack of awareness of new or worsening symptoms, medical conditions such as heart failure might not have become known to physicians without continued oversight until a subsequent visit, possibly resulting in untoward outcomes.

Although multiple evidence-based cardiac lifestyle resources exist, they are not integrated and lack a personalized lifestyle approach that takes into account individual preferences and whose progress is driven by the patient. For example, with regard to medication adherence, one participant joked that he forgets to take his medications sometimes, yet never forgets to walk his dog. Thus, a real-world strategy that worked was having his medication box placed right next to the dog leash.

A separate study of AF patients (n = 140) at CUMC by a member of our team found significant gaps in knowledge, both about AF in general and specifically about approaches for reducing AF risk factors (dissertation research; manuscripts in progress). For instance, 30% of patients did not know common AF triggers (eg, alcohol); 37% did not understand the rationale for taking anticoagulants; and 35% did not understand the risks associated with asymptomatic AF. Therefore, patient education and self-management guidance via a nurse-led intervention may be a medium for AF cardiac risk factor education and highlights the unique and innovative aspect of the ACTION intervention. Some evidence suggests that lifestyle intervention strategies with more frequent participant contact generally yield better outcomes,^66,67^ and personalized lifestyle modification with a “human component” is important in creating social support to motivate individuals to change their health behaviors.^68,69^ This observation is similar to our findings, wherein the majority of the participants reported the nurse as the most essential of the intervention components “that keep them motivated” in working toward their lifestyle health goals.

Likewise, researchers have also shown that human support is the most important component in the effectiveness of and adherence to behavior change interventions. Thoughtfully introducing a digital person-to-person component to replace clinic visits can provide the needed human support while diminishing the barriers of in-person meetings.^69^ In fact, many patients report missing human interaction when clinical visits are totally replaced by technology, suggesting that it is an essential component of patient self-management.^70^ While motivational interviewing has been successful for cardiac lifestyle modifications,^71–74^ using a multicomponent mHealth cardiac lifestyle intervention that includes motivational interviewing with AF patients was an innovative aspect of our research study. Using motivational interviewing, the cardiac nurse could tailor the Life’s Simple 7^®^ and My AFib Experience™ AFib Symptom Tracker web-based resources (American Heart Association, Dallas, TX, USA), discuss AF and cardiac lifestyle health goals, and provide emotional support to faciliate positive behavior changes. In fact, 96% of the participants reported the ACTION intervention to be useful in facilitating and sustaining behavior changes and in “staying motivated.” Finally, our study results are similar to those of previous research that demonstrated that improving cardiac lifestyle^75^ reduces AF recurrence and symptoms^47,76^ and that mHealth combined with human support is appropriate to promote lifestyle changes.^77–83^

Data from the ICM (eg, frequency, duration, ECG pattern of AF) allowed for the identification of AF and its triggers and symptoms at the time of AF occurrence. The ECG transmission presented in **Figure 3** captured AF in the setting of the symptom of shortness of breath. In addition, this participant reported a stressful day at work and that he forgot to take his β-blocker on the day of the transmission. The additional data from the ICM allowed us to improve our understanding of the associations between the presence or absence of individual self-reported symptoms and medication adherence with β-blockers and antiarrhythmics. In this subgroup of participants, we were able to more precisely correlate symptoms and true AF events with additional details such as the time of day and potential triggers reported by the participant (eg, exercise, lack of sleep, stress) via the ICM.

Recent updates to the AF guidelines highlight adherence to prescribed AF treatments, especially anticoagulation, to be of the utmost importance in order to avoid AF-associated complications such as stroke.^84^ In the Telephone Contacts to Improve Adherence to Dual Antiplatelet Therapy Following Drug-eluting Stent Implantation (EASY-IMPACT) trial, telephone support (ie, four telephone calls from a nurse) dramatically improved sustained medication adherence rates in patients who had received drug-eluting stents. After the first year, more than 90% of the patients who had received standard-of-care follow-up were adherent with their prescribed medication, whereas more than 99% of the patients who received telephone follow-up were adherent,^85^ highlighting the impact of nurse-led interventions in improving medication adherence in cardiovascular populations. Finally, a small nurse-led pilot study conducted in Australia examined the use of an educational intervention at the time of AF ablation and found a positive effect on improving symptom recognition. Similar to the results of their work, we found that shortness of breath, exercise intolerance, and fatigue were common in our cohort as well.^86^

In addition, nurse-led heart failure clinics have been shown to improve survival and self-care in patients with heart failure.^87^ In a prospective randomized trial, patients assigned to a nurse-led cardiac clinic had fewer deaths, hospital admissions, and days in the hospital as well as higher self-care scores at both three months and 12 months.^87^ Separately, the Australian Nurse-led Intervention for Less Chronic Heart Failure (NIL-CHF) study showed that the nurse-led interventions were associated with better cardiac recovery as well as reduced emergency and unplanned readmission costs.^88,89^ This study differed from the present ACTION intervention in that it focused on heart failure and involved a nurse-led home- and clinic-based program as opposed to a telehealth intervention. ACTION was not primarily focused on heart failure and was instead primarily focused on an AF population with multiple cardiac risk factors. Moreover, the ACTION intervention’s focus was on using motivational interviewing to encourage lifestyle modification, whereas the nurse-led interventions for heart failure, a chronic condition, are focused on the modification of therapy and hospital admission suggestions.^90^

### Study limitations

Although the above results are encouraging, some limitations do exist in our single-arm pilot study. For example, the sample size was small, there was no control group, and the study was conducted at a single center. Furthermore, although participants noted working with a nurse helped them to stay motivated on how to live with AF and maintain a healthy lifestyle, the association of underlying cardiac risk factors with AF over time was not systematically captured. In addition, the long-term effects of the nurse-led intervention on medication adherence and the burden of AF and associated symptoms remain unknown.

## Conclusion

Findings from this pilot study provide initial evidence supporting a nurse-led lifestyle intervention delivered in a real-world setting and address the broader goals of the National Institutes of Health and Healthy People 2020 initiative to reduce morbidity and mortality.^91,92^ Knowledge from this pilot study supports the need to test the ACTION intervention in a larger AF cohort. This could ultimately provide further evidence to inform the existing AF guidelines about the impact of lifestyle modification on reducing AF burden and could be easily transferable into multiple settings and be widely adopted and disseminated in clinical practice to improve cardiac health and reduce the burden of AF in society in the future.

### Future directions

It remains unknown as to what the optimal intervention frequency should be for AF patients to achieve quantifiable sustained improvements in cardiac health outcomes. The current study provides initial evidence for a larger randomized controlled trial to evaluate the efficacy and determine the optimal dose of a nurse-led lifestyle coaching intervention and contact frequency (ie, weekly, biweekly, or monthly) for AF patients to improve symptom recognition and cardiac health outcomes using a personalized approach. In addition, further studies are needed to examine the role of sex, age, ethnicity, and engagement with mHealth on the long-term effects of a nurse-led intervention for improving cardiac health, medication adherence, reducing symptom burden, rehospitalization rates, and cost.

## Figures and Tables

**Table 1:** Patient Demographics

Parameter	Patient Data
Age (mean ± standard deviation)	59 ± 11 years
Male gender	76%
Race	
White	60%
Hispanic	29%
Black	3%
One or more races	8%
Clinical history	
Overweight/obese	79%
Hypertension	62%
Hyperlipidemia	61%
Physically inactive	52%
One or more of the Life’s Simple 7^®^ measures identified at baseline*	88%

*Life’s Simple 7^®^ is a web-based resource of the American Heart Association (Dallas, TX, USA).

## References

[1] Freedman B, Camm J, Calkins H (2017). Screening for atrial fibrillation: a report of the AF-SCREEN international collaboration.. Circulation..

[2] Roger VL, Go AS, Lloyd-Jones DM (2012). Heart disease and stroke statistics—2012 update: a report from the American Heart Association.. Circulation..

[3] Kim MH, Johnston SS, Chu BC, Dalal MR, Schulman KL (2011). Estimation of total incremental health care costs in patients with atrial fibrillation in the United States.. Circ Cardiovasc Qual Outcomes..

[4] Wang TJ, Massaro JM, Levy D (2003). A risk score for predicting stroke or death in individuals with new-onset atrial fibrillation in the community: the Framingham Heart Study.. JAMA..

[5] Stewart S, Hart CL, Hole DJ, McMurray JJ (2002). A population-based study of the long-term risks associated with atrial fibrillation: 20-year follow-up of the Renfrew/Paisley study.. Am J Med..

[6] Lip GY, Nieuwlaat R, Pisters R, Lane DA, Crijns HJ (2010). Refining clinical risk stratification for predicting stroke and thromboembolism in atrial fibrillation using a novel risk factor-based approach: the Euro heart survey on atrial fibrillation.. Chest..

[7] Wolf PA, Abbott RD, Kannel WB (1991). Atrial fibrillation as an independent risk factor for stroke: the Framingham Study.. Stroke..

[8] Miyasaka Y, Barnes ME, Gersh BJ (2006). Incidence and mortality risk of congestive heart failure in atrial fibrillation patients: a community-based study over two decades.. Eur Heart J..

[9] Aleong RG, Sauer WH, Davis G, Bristow MR (2014). New-onset atrial fibrillation predicts heart failure progression.. Am J Med..

[10] Soliman EZ, Safford MM, Muntner P (2014). Atrial fibrillation and the risk of myocardial infarction.. JAMA Intern Med..

[11] Piccini JP, Hammill BG, Sinner MF (2012). Incidence and prevalence of atrial fibrillation and associated mortality among Medicare beneficiaries, 1993-2007.. Circ Cardiovasc Qual Outcomes..

[12] Miyasaka Y, Barnes ME, Bailey KR (2007). Mortality trends in patients diagnosed with first atrial fibrillation: a 21-year community-based study.. J Am Coll Cardiol..

[13] Benjamin EJ, Wolf PA, D’Agostino RB, Silbershatz H, Kannel WB, Levy D (1998). Impact of atrial fibrillation on the risk of death: the Framingham Heart Study.. Circulation..

[14] Wolowacz SE, Samuel M, Brennan VK, Jasso-Mosqueda JG, Van Gelder IC (2011). The cost of illness of atrial fibrillation: a systematic review of the recent literature.. Europace..

[15] Garg PK, O’Neal WT, Ogunsua A (2018). Usefulness of the American Heart Association’s Life Simple 7 to Predict the Risk of Atrial Fibrillation (from the Reasons for Geographic and Racial Differences in Stroke [REGARDS] study).. Am J Cardiol..

[16] Luderitz B, Jung W (2000). Quality of life in patients with atrial fibrillation.. Arch Intern Med..

[17] January CT, Wann LS, Alpert JS (2014). 2014 AHA/ACC/HRS guideline for the management of patients with atrial fibrillation: executive summary: a report of the American College of Cardiology/American Heart Association Task Force on practice guidelines and the Heart Rhythm Society.. Circulation..

[18] Fuster V, Ryden LE, Cannom DS (2011). 2011 ACCF/AHA/HRS focused updates incorporated into the ACC/AHA/ESC 2006 Guidelines for the management of patients with atrial fibrillation: a report of the American College of Cardiology Foundation/American Heart Association Task Force on Practice Guidelines developed in partnership with the European Society of Cardiology and in collaboration with the European Heart Rhythm Association and the Heart Rhythm Society.. J Am Coll Cardiol..

[19] Morady F (1999). Radio-frequency ablation as treatment for cardiac arrhythmias.. N Engl J Med..

[20] Bubien RS, Knotts-Dolson SM, Plumb VJ, Kay GN (1996). Effect of radiofrequency catheter ablation on health-related quality of life and activities of daily living in patients with recurrent arrhythmias.. Circulation..

[21] Kuck KH, Brugada J, Furnkranz A (2016). Cryoballoon or radiofrequency ablation for paroxysmal atrial fibrillation.. N Engl J Med..

[22] Calkins H, Kuck KH, Cappato R (2012). 2012 HRS/EHRA/ECAS Expert Consensus Statement on Catheter and Surgical Ablation of Atrial Fibrillation: recommendations for patient selection, procedural techniques, patient management and follow-up, definitions, endpoints, and research trial design.. Europace..

[23] Darby AE (2016). Recurrent atrial fibrillation after catheter ablation: considerations for repeat ablation and strategies to optimize success.. J Atr Fibrillation..

[24] Sultan A, Luker J, Andresen D (2017). Predictors of atrial fibrillation recurrence after catheter ablation: data from the German Ablation Registry.. Sci Rep..

[25] Alcaraz R, Hornero F, Rieta JJ (2011). Noninvasive time and frequency predictors of long-standing atrial fibrillation early recurrence after electrical cardioversion.. Pacing Clin Electrophysiol..

[26] Elesber AA, Rosales AG, Herges RM (2006). Relapse and mortality following cardioversion of new-onset vs. recurrent atrial fibrillation and atrial flutter in the elderly.. Eur Heart J..

[27] Benjamin EJ, Chen PS, Bild DE (2009). Prevention of atrial fibrillation: report from a national heart, lung, and blood institute workshop.. Circulation..

[28] McCabe PJ, Schad S, Hampton A, Holland DE (2008). Knowledge and self-management behaviors of patients with recently detected atrial fibrillation.. Heart Lung..

[29] Nalliah CJ, Sanders P, Kottkamp H, Kalman JM (2016). The role of obesity in atrial fibrillation.. Eur Heart J..

[30] Huxley RR, Lopez FL, Folsom AR (2011). Absolute and attributable risks of atrial fibrillation in relation to optimal and borderline risk factors: the Atherosclerosis Risk in Communities (ARIC) study.. Circulation..

[31] Alonso A, Krijthe BP, Aspelund T (2013). Simple risk model predicts incidence of atrial fibrillation in a racially and geographically diverse population: the CHARGE-AF consortium.. J Am Heart Assoc..

[32] Whelton PK, Carey RM, Aronow WS (2018). 2017 ACC/AHA/AAPA/ABC/ACPM/AGS/APhA/ASH/ASPC/NMA/PCNA Guideline for the Prevention, Detection, Evaluation, and Management of High Blood Pressure in Adults: Executive Summary: A Report of the American College of Cardiology/American Heart Association Task Force on Clinical Practice Guidelines.. J Am Coll Cardiol..

[33] Voskoboinik A, Prabhu S, Ling LH, Kalman JM, Kistler PM (2016). Alcohol and atrial fibrillation: a sobering review.. J Am Coll Cardiol..

[34] Larsson SC, Drca N, Wolk A (2014). Alcohol consumption and risk of atrial fibrillation: a prospective study and dose-response meta-analysis.. J Am Coll Cardiol..

[35] Benjamin EJ, Levy D, Vaziri SM, D’Agostino RB, Belanger AJ, Wolf PA (1994). Independent risk factors for atrial fibrillation in a population-based cohort. The Framingham Heart Study.. JAMA..

[36] Wang TJ, Parise H, Levy D (2004). Obesity and the risk of new-onset atrial fibrillation.. JAMA..

[37] Wong CX, Sullivan T, Sun MT (2015). Obesity and the risk of incident, post-operative, and post-ablation atrial fibrillation. A meta-analysis of 626,603 individuals in 51 studies.. JACC Clin Electrophysiol..

[38] Thomas MC, Dublin S, Kaplan RC (2008). Blood pressure control and risk of incident atrial fibrillation.. Am J Hypertens..

[39] Chen LY, Bigger JT, Hickey KT (2016). Effect of intensive blood pressure lowering on incident atrial fibrillation and P-wave indices in the ACCORD blood pressure trial.. Am J Hypertens..

[40] Jamaly S, Carlsson L, Peltonen M, Jacobson P, Sjostrom L, Karason K (2016). Bariatric surgery and the risk of new-onset atrial fibrillation in Swedish obese subjects.. J Am Coll Cardiol..

[41] Pathak RK, Evans M, Middeldorp ME (2017). Cost-effectiveness and clinical effectiveness of the risk factor management clinic in atrial fibrillation. The CENT Study.. JACC Clin Electrophysiol..

[42] Hong KL, Glover BM (2018). The impact of lifestyle intervention on atrial fibrillation.. Curr Opin Cardiol..

[43] Abed HS, Wittert GA, Leong DP (2013). Effect of weight reduction and cardiometabolic risk factor management on symptom burden and severity in patients with atrial fibrillation: a randomized clinical trial.. JAMA..

[44] Steinberg JS, Palekar R, Sichrovsky T (2014). Very long-term outcome after initially successful catheter ablation of atrial fibrillation.. Heart Rhythm..

[45] Lu ZH, Liu N, Bai R (2015). HbA1c levels as predictors of ablation outcome in type 2 diabetes mellitus and paroxysmal atrial fibrillation.. Herz..

[46] Whelton PK, Carey RM, Aronow WS (2018). 2017 ACC/AHA/AAPA/ABC/ACPM/AGS/APhA/ASH/ASPC/NMA/PCNA Guideline for the Prevention, Detection, Evaluation, and Management of High Blood Pressure in Adults: A Report of the American College of Cardiology/American Heart Association Task Force on Clinical Practice Guidelines.. J Am Coll Cardiol..

[47] Pathak RK, Middeldorp ME, Lau DH (2014). Aggressive risk factor reduction study for atrial fibrillation and implications for the outcome of ablation: the ARREST-AF cohort study.. J Am Coll Cardiol..

[48] Miller WR, Rollnick S (2002). Motivational Interviewing: Preparing People to Change Addictive Behavior..

[49] Rollnick S, Miller WR, Butler CC (2008). Motivational Interviewing in Health Care..

[50] Miller WR, Rollnick S (1991). Motivational Interviewing: Preparing People to Change Addictive Behavior..

[51] Resnicow K, DiIorio C, Soet JE, Borrelli B, Hecht J, Ernst D (2002). Motivational interviewing in health promotion: It sounds like something is changing.. Health Psychol..

[52] Lee WWM, Choi KC, Yum RWY, Yu DSF, Chair SY (2016). Effectiveness of motivational interviewing on lifestyle modification and health outcomes of clients at risk or diagnosed with cardiovascular diseases: a systematic review.. Int J Nurs Stud..

[53] Fisher JD, Fisher WA, Amico KR, Harman JJ (2006). An information-motivation-behavioral skills model of adherence to antiretroviral therapy.. Health Psychol..

[54] Fein AS, Shvilkin A, Shah D (2013). Treatment of obstructive sleep apnea reduces the risk of atrial fibrillation recurrence after catheter ablation.. J Am Coll Cardiol..

[55] Malmo V, Nes BM, Amundsen BH (2016). Aerobic interval training reduces the burden of atrial fibrillation in the short term: a randomized trial.. Circulation..

[56] Bowyer JL, Tully PJ, Ganesan AN, Chahadi FK, Singleton CB, McGavigan AD (2017). A randomised controlled trial on the effect of nurse-led educational intervention at the time of catheter ablation for atrial fibrillation on quality of life, symptom severity and rehospitalisation.. Heart Lung Circ..

[57] American Heart Association. Symptom Tracker..

[58] Ammentorp J, Uhrenfeldt L, Angel F, Ehrensvard M, Carlsen EB, Kofoed PE (2013). Can life coaching improve health outcomes?—a systematic review of intervention studies.. BMC Health Serv Res..

[59] Vale MJ, Jelinek MV, Best JD (2003). Coaching patients On Achieving Cardiovascular Health (COACH): a multicenter randomized trial in patients with coronary heart disease.. Arch Intern Med..

[60] Thacker EL, Gillett SR, Wadley VG (2014). The American Heart Association Life’s Simple 7 and incident cognitive impairment: the Reasons for Geographic and Racial Differences in Stroke (REGARDS) study.. J Am Heart Assoc..

[61] Estes NAM, Sacco RL, Al-Khatib SM (2011). American Heart Association atrial fibrillation research summit: a conference report from the American Heart Association.. Circulation..

[62] Lau DH, Nattel S, Kalman JM, Sanders P (2017). Modifiable risk factors and atrial fibrillation.. Circulation..

[63] Hendriks JM, de Wit R, Crijns HJ (2012). Nurse-led care vs. usual care for patients with atrial fibrillation: results of a randomized trial of integrated chronic care vs. routine clinical care in ambulatory patients with atrial fibrillation.. Eur Heart J..

[64] Low KG, Giasson H, Connors S, Freeman D, Weiss R (2013). Testing the effectiveness of motivational interviewing as a weight reduction strategy for obese cardiac patients: a pilot study.. Int J Behav Med..

[65] Fraiche AM, Eapen ZJ, McClellan MB (2017). Moving beyond the walls of the clinic: opportunities and challenges to the future of telehealth in heart failure.. JACC Heart Fail..

[66] Baillot A, Romain AJ, Boisvert-Vigneault K (2015). Effects of lifestyle interventions that include a physical activity component in class II and III obese individuals: a systematic review and meta-analysis.. PLoS One..

[67] Lv N, Azar KMJ, Rosas LG, Wulfovich S, Xiao L, Ma J (2017). Behavioral lifestyle interventions for moderate and severe obesity: a systematic review.. Prev Med..

[68] Forbes CC, Plotnikoff RC, Courneya KS, Boule NG (2010). Physical activity preferences and type 2 diabetes: exploring demographic, cognitive, and behavioral differences.. Diabetes Educ..

[69] Santarossa S, Kane D (2018). Exploring the role of in-person components for online health behavior change interventions: can a digital person-to-person component suffice?.. J Med Internet Res..

[70] Sanger PC, Hartzler A, Lordon RJ (2016). A patient-centered system in a provider-centered world: challenges of incorporating post-discharge wound data into practice.. J Am Med Inform Assoc..

[71] Artinian NT, Fletcher GF, Mozaffarian D (2010). Interventions to promote physical activity and dietary lifestyle changes for cardiovascular risk factor reduction in adults: a scientific statement from the American Heart Association.. Circulation..

[72] Koelewijn-van Loon MS, van Steenkiste B, Ronda G (2008). Improving patient adherence to lifestyle advice (IMPALA): a cluster-randomised controlled trial on the implementation of a nurse-led intervention for cardiovascular risk management in primary care (protocol).. BMC Health Serv Res..

[73] Lee WW, Choi KC, Yum RW, Yu DS, Chair SY (2016). Effectiveness of motivational interviewing on lifestyle modification and health outcomes of clients at risk or diagnosed with cardiovascular diseases: a systematic review.. Int J Nurs Stud..

[74] Bredie SJ, Fouwels AJ, Wollersheim H, Schippers GM (2011). Effectiveness of nurse-based motivational interviewing for smoking cessation in high risk cardiovascular outpatients: a randomized trial.. Eur J Cardiovasc Nurs..

[75] Calkins H, Brugada J, Packer DL (2007). HRS/EHRA/ECAS expert Consensus Statement on catheter and surgical ablation of atrial fibrillation: recommendations for personnel, policy, procedures and follow-up. A report of the Heart Rhythm Society (HRS) Task Force on catheter and surgical ablation of atrial fibrillation.. Heart Rhythm..

[76] Pathak RK, Middeldorp ME, Meredith M (2015). Long-term effect of goal-directed weight management in an atrial fibrillation cohort: a long-term follow-up study (LEGACY).. J Am Coll Cardiol..

[77] Bakken S, Grullon-Figueroa L, Izquierdo R (2006). Development, validation, and use of English and Spanish versions of the telemedicine satisfaction and usefulness questionnaire.. J Am Med Inform Assoc..

[78] Free C, Phillips G, Galli L (2013). The effectiveness of mobile-health technology-based health behaviour change or disease management interventions for health care consumers: a systematic review.. PLoS Med..

[79] Kitsiou S, Pare G (2015). Effects of home telemonitoring interventions on patients with chronic heart failure: an overview of systematic reviews.. J Med Internet Res..

[80] Patel R, Chang T, Greysen SR, Chopra V (2015). Social media use in chronic disease: a systematic review and novel taxonomy.. Am J Med..

[81] Hou C, Carter B, Hewitt J, Francisa T, Mayor S (2016). Do mobile phone applications improve glycemic control (HbA1c) in the self-management of diabetes?. A systematic review, meta-analysis, and GRADE of 14 randomized trials.. Diabetes Care..

[82] Gorst SL, Armitage CJ, Brownsell S, Hawley MS (2014). Home telehealth uptake and continued use among heart failure and chronic obstructive pulmonary disease patients: a systematic review.. Ann Behav Med..

[83] Liu L, Stroulia E, Nikolaidis I, Miguel-Cruz A, Rios Rincon A (2016). Smart homes and home health monitoring technologies for older adults: a systematic review.. Int J Med Inform..

[84] Camm AJ, Lip GY, De Caterina R (2012). 2012 focused update of the ESC guidelines for the management of atrial fibrillation: an update of the 2010 ESC Guidelines for the management of atrial fibrillation. Developed with the special contribution of the European Heart Rhythm Association.. Eur Heart J..

[85] Rinfret S, Rodes-Cabau J, Bagur R (2013). Telephone contact to improve adherence to dual antiplatelet therapy after drug-eluting stent implantation.. Heart..

[86] Stewart S, Ball J, Horowitz JD (2015). Standard versus atrial fibrillation-specific management strategy (SAFETY) to reduce recurrent admission and prolong survival: pragmatic, multicentre, randomised controlled trial.. Lancet..

[87] Stromberg A, Martensson J, Fridlund B, Levin LA, Karlsson JE, Dahlstrom U (2003). Nurse-led heart failure clinics improve survival and self-care behaviour in patients with heart failure: results from a prospective, randomised trial.. Eur Heart J..

[88] Stewart S, Chan YK, Wong C (2015). Impact of a nurse-led home and clinic-based secondary prevention programme to prevent progressive cardiac dysfunction in high-risk individuals: the nurse-led Intervention for Less Chronic Heart Failure (NIL-CHF) randomized controlled study.. Eur J Heart Fail..

[89] Maru S, Byrnes J, Carrington MJ (2018). Economic evaluation of a nurse-led home and clinic-based secondary prevention programme to prevent progressive cardiac dysfunction in high-risk individuals: the Nurse-led Intervention for Less Chronic Heart Failure (NIL-CHF) randomized controlled study.. Eur J Cardiovasc Nurs..

[90] Scalvini S, Zanelli E, Volterrani M (2004). A pilot study of nurse-led, home-based telecardiology for patients with chronic heart failure.. J Telemed Telecare..

[91] Lloyd-Jones DM, Hong Y, Labarthe D (2010). Defining and setting national goals for cardiovascular health promotion and disease reduction: the American Heart Association’s strategic impact goal through 2020 and beyond.. Circulation..

[92] Institute of Medicine, Board on Health Care Services, Committee on Comparative Effectiveness Research Prioritization. (2009). Initial National Priorities for Comparative Effectiveness Research..

